# Protected butyrate coating systems affect pellet quality and the growth performance, intestinal morphology, and meat quality of broilers fed diets containing poultry by-product meal

**DOI:** 10.1016/j.vas.2026.100932

**Published:** 2026-11-04

**Authors:** Seyed Ghasem Shirangi, Mahmoud Shams Shragh, Behrouz Dastar, Mohammad Hossein Mohammadi, Enayat Rahmatnejad

**Affiliations:** a Department of Animal and Poultry Nutrition, Faculty of Animal Science, Gorgan University of Agricultural Sciences and Natural Resources, 4918943464, Gorgan, Iran; b Department of Animal Science, College of Agriculture and Natural Resources, University of Tehran, P. O. Box 31585-4111, Karaj, Iran; c Department of Animal Science, Faculty of Agriculture and Natural Resources, Persian Gulf University, Bushehr, 75169, Iran

**Keywords:** Broiler, Protected butyrate, Growth performance, Gut morphology, Meat quality

## Abstract

This study evaluated four protected butyrate (PB) sources for growth, carcass traits, intestinal morphology, serum biochemistry, meat quality, and physical pellet quality (PPQ) in broilers fed diet containing poultry by-product meal (PBM). 300 Indian River chicks were assigned to five treatments in a completely randomized design for 42 d. Birds received a control diet or 300 ppm PB as PB-A (ButiPEARL®), PB-B (Eudragit® L100-coated), PB-C (saturated fat-coated), or PB-D (saturated fat- and Tween 80-coated). PB-C reduced feed intake (FI) and body weight gain during the starter and grower phases (*P* < 0.01), while overall FI was lower in PB-C than in PB-B (*P* < 0.05). All PB forms numerically improved starter feed conversion ratio (FCR), with PB-B having lower FCR than the control (*P* = 0.01). Abdominal fat pad was higher in PB-C than in PB-A and PB-B (*P* < 0.05). In the duodenum, PB-B increased villus height and villus height to crypt depth ratio (*P* < 0.05), while PB-D increased villus width and villus surface area relative to the control (*P* < 0.05). Jejunal and ileal morphology and serum biochemical indices were unaffected (*P* > 0.05). PB-A, PB-B, and PB-D increased muscle water-holding capacity relative to PB-C, whereas PB-B and PB-D reduced cooking loss compared with the control (*P* < 0.01). PB-A, PB-C, and PB-D increased pellet durability relative to the control and PB-B (*P* < 0.001). Overall, PB efficacy was coating-dependent, PB-B most consistently improved starter feed efficiency, duodenal morphology, and meat quality, whereas lipid-based PB enhanced PPQ.

## Highlights

• Protected butyrate responses depended on coating material and delivery system.
• Eudragit L100-coated butyrate improved feed efficiency during the starter phase.
• Protected butyrate enhanced duodenal morphology.
• Protected butyrate forms improved meat quality.
• Lipid-coated butyrate improved pellet durability.

## Graphical abstract

**Figure:**
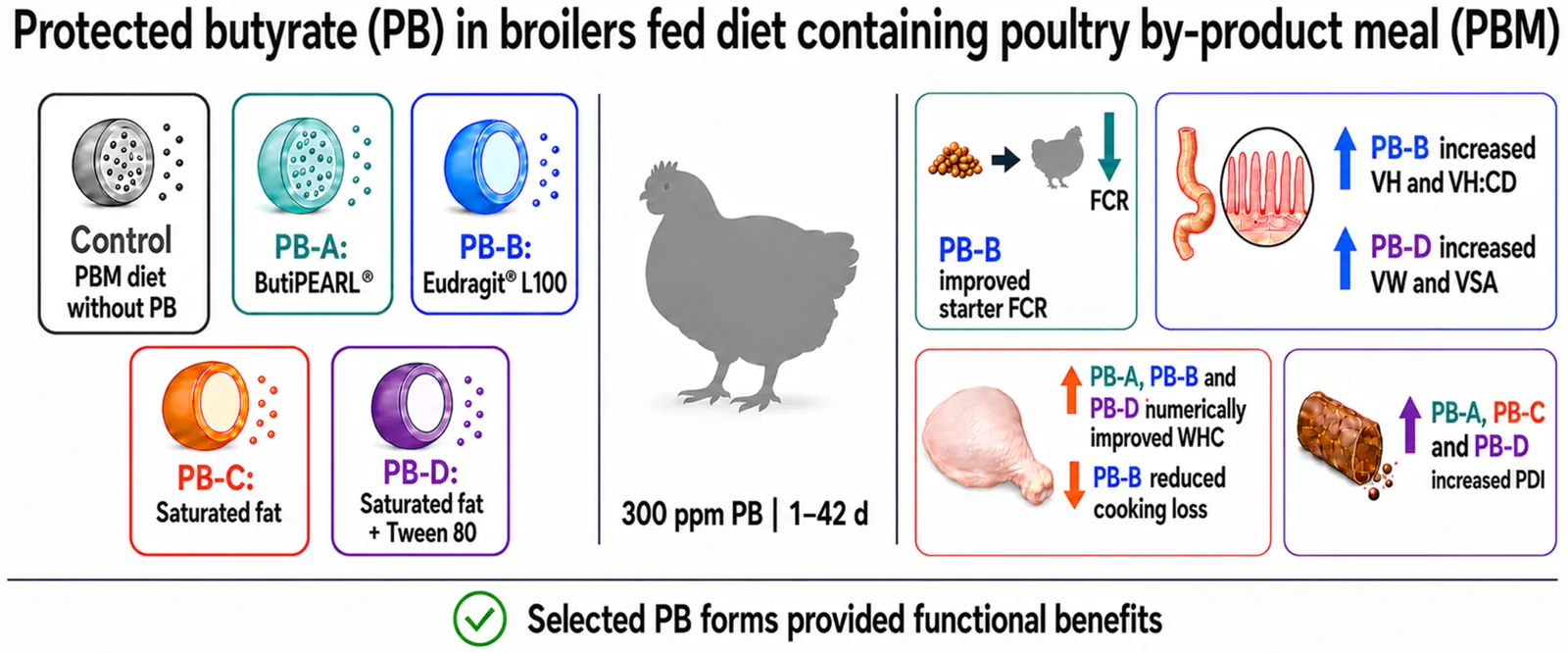

## 1 Introduction

The continued expansion of poultry meat production has increased the demand for feed ingredients that are both economically viable and environmentally sustainable. Broiler diets are commonly formulated with maize as the primary energy source and soybean meal (SBM) as the principal protein source because of its favourable amino acid profile. However, reliance on SBM raises concerns regarding price volatility, supply-chain disruptions, and environmental impacts such as deforestation and land-use change. Consequently, alternative protein sources have received increasing attention in broiler nutrition (Vlaicu et al., 2026).

Poultry by-product meal (PBM) is a protein-rich feed ingredient produced from rendered materials generated during poultry processing, including heads, feet, and internal organs (Ahmad et al., 2024). Despite its nutritional and economic value, variation in composition and nutrient digestibility may limit its efficient utilization in broiler diets (Frempong et al., 2019; Volpato et al., 2022). Therefore, nutritional strategies that improve gastrointestinal function and nutrient utilization may enhance the feeding value of PBM-containing diets.

Butyrate is a short-chain fatty acid that supports intestinal health by providing energy to epithelial cells and contributing to barrier integrity, microbial balance, and intestinal morphology (Aljumaah et al., 2020; Mátis et al., 2022). However, unprotected butyric acid is rapidly absorbed in the upper gastrointestinal tract, limiting its availability in more distal intestinal regions. Coating or encapsulation can control its release and increase the amount delivered to the intended intestinal site (El-Saadony et al., 2022; Ghasemi et al., 2025). Several studies have demonstrated that protected butyrate (PB) can positively influence growth performance, intestinal morphology, and immune responses in broiler chickens (Ghasemi et al., 2025). Furthermore, physical pellet quality is influenced by feed formulation, particularly the type and level of protein, fiber, and fat, and may therefore vary according to the composition and coating material of PB products (Mohammadi Ghasem Abadi et al., 2019; Moradi et al., 2024).

The delivery characteristics of protected butyrate are determined largely by the coating material. Lipid matrices provide physical protection through their hydrophobic properties, whereas Eudragit® L100 is a pH-sensitive methacrylic copolymer that remains stable under acidic conditions and dissolves at the higher pH of the intestine (Jain et al., 2020; Sester et al., 2020). Tween 80 (Polysorbate 80) is a non-ionic surfactant that serves as an emulsifier, providing better solubility and homogeneity of coating agents and improving coating effectiveness (Ameta et al., 2023).

Importantly, limited information is available regarding the comparative efficacy of different PB delivery systems in broilers fed PBM-containing diets. Because coating composition may influence the intestinal release of butyrate, a direct comparison of different PB formulations is warranted. Therefore, this study evaluated the effects of different PB forms on growth performance, carcass traits, intestinal morphology, serum biochemical parameters, meat quality, and PPQ in broiler chickens fed diets containing PBM. We hypothesized that the biological responses of broilers and the physical quality of the pellets would differ according to the coating system used for PB.

## 2 Materials and methods

### 2.1 Preparation of protected butyrate (PB)

Four different forms of protected butyrate (PB) were used in the present study. All products were calcium-based butyrate sources. PB-A was a commercial product (ButiPEARL®, Kemin, Belgium) and served as the reference source. The PB-A contained 50 % butyric acid calcium salt, 49.9 % hydrogenated vegetable oils (palm), and 0.1 % flavouring compound (vanillin). PB-B, PB-C, and PB-D were prepared using different encapsulation and processing techniques to modify the release characteristics of calcium butyrate, and, like PB-A, all contained 50% calcium butyrate (Asia Fara Gene Co., Iran).

PB-B consisted of calcium butyrate encapsulated with the Eudragit® L100 100 copolymer using a fluidized-bed coating technique. In summary, 60 grams of calcium butyrate salt were charged to the fluidized-bed coating pan, and coating medium consisting of 6% and 4% w/v of Eudragit® L100 and ethyl cellulose in water-ethanol blend (70:30, v/v) was applied at the flow rate of 3 mL/min while maintaining a nozzle pressure of 1.2 bar. Triethyl citrate was added as a plasticizer at the ratio of 20% w/w with respect to the total polymer weight. Inlet air temperature was kept constant at 40°C and coating time was set to 15 minutes, with the product being dried to less than 2% moisture content using freeze-drying (Li et al., 2013; Zhang et al., 2020). PB-C was produced by spheronization of calcium butyrate, where the salt was premixed at a 1:1 (w/w) ratio with a saturated fat powder containing 70% palmitic acid, followed by processing in a extruder at 40-50°C and 200 rpm. The resulting product was cooled with water, milled, and used as a 50% premix (Huang et al., 2014; Onrust et al., 2020). PB-D was prepared using a combined encapsulation-emulsion technique, in which calcium butyrate was mixed at a 1:1 (w/w) ratio with a saturated fat powder containing 70% palmitic acid and Tween 80 in a homogenizer for 10 min, followed by extrusion at 50-65°C and 150 rpm. The final product was cooled, freeze-dried, and milled prior to use (Ghasemi et al., 2018).

The morphological characteristics of the different protected butyrate (PB) forms were examined using light microscopy. Samples of PB-A, PB-B, PB-C, and PB-D were observed under a light microscope to evaluate particle shape, surface structure, and general morphological differences resulting from the different encapsulation techniques (Fig. 1).

**Fig. 1:**
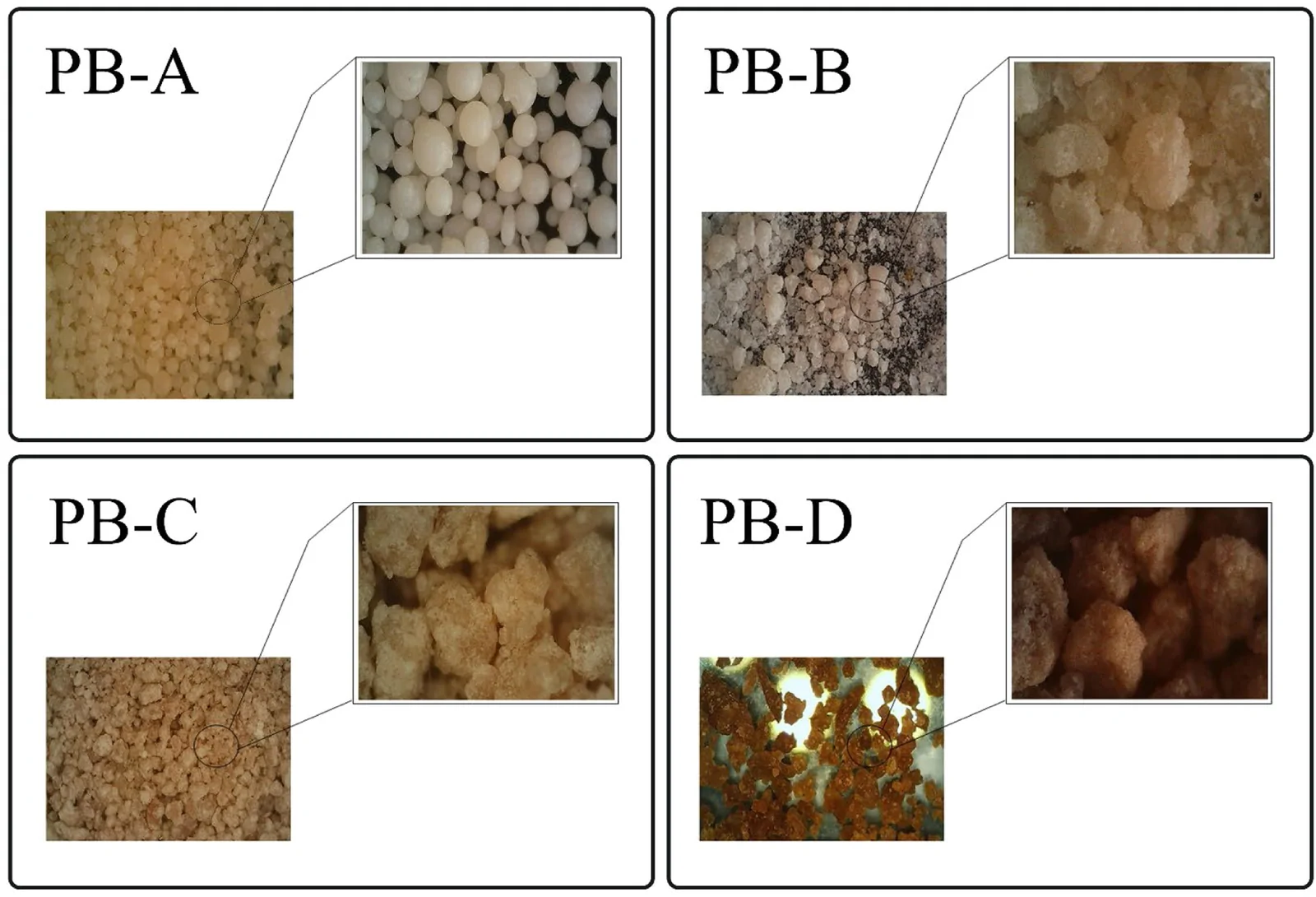
Light microscopic images of different protected butyrate. Abbreviations: **PB-A,** protected butyrate ButiPEARL®, Kemin; **PB-B,** butyrate protected with Eudragit® L100 copolymer; **PB-C,** butyrate protected with saturated fat; **PB-D,** butyrate protected with saturated fat and Tween 80.

### 2.2 Birds and experimental design

Three hundred one-day-old male Indian River broiler chicks were distributed among five dietary treatments according to a completely randomized design, with five replicate pens per treatment. At placement, all chicks were weighed individually and allocated to pens so that each replicate consisted of 12 birds with a similar initial body weight (41 ± 0.6 g). The experiment lasted for 42 days. The birds were offered either a basal diet (control) or a basal diet fortified with 300 ppm of PB-A, PB-B, PB-C, or PB-D. Each PB product was included at 300 mg/kg of complete diet. Because the products contained 50% (w/w) calcium butyrate, this inclusion rate supplied 150 mg of calcium butyrate per kilogram of diet. The experiment involved a three-stage feed program including starter (1-14 days), grower (15-28 days), and finisher (29-42 days) diets. The basal diets were formulated based on the nutrient recommendations for Indian River broilers, with minor modifications (Table 1). The PBM used in this study was obtained from Sefid Zarbal Golestan Co. (Bandar Torkaman, Iran). Before diet formulation, its chemical composition was analyzed based on AOAC (2007) and found to contain 98.91% dry mater, 48.3% crude protein, 2.13% crude fiber, 19.5% ether extract, and 5.71% ash. All experimental diets were prepared in pelleted form.

**Table 1:** Ingredient and nutrient composition of basal diets (%, unless stated otherwise).

Item	Starter (d 1-14)	Grower (d 15-28)	Finisher (d 29-42)
**Ingredients**			
Corn (CP = 7.3 %)	53.56	60.60	65.56
Soybean Meal (CP = 44 %)	32.00	25.60	21.00
Poultry By-product (CP = 48.3 %)	10	10	10
Soybean Oil	1.5	1.5	1.5
DL- Methionine	0.400	0.370	0.340
L- Lysine HCl	0.330	0.320	0.315
L- Threonine	0.165	0.147	0.130
Dicalcium Phosphate	0.920	0.547	0.265
Limestone	0.275	0.070	0.045
Sodium Bicarbonate	0.265	0.260	0.260
Vitamin and Mineral Premix 1	0.200	0.200	0.200
Choline HCL	0.050	0.050	0.050
Toxin Binder	0.050	0.050	0.050
Coccidiostat 2	0.050	0.050	0.050
Phytase 3	0.005	0.005	0.005
Pellet Binder 4	0.200	0.200	0.200
Sand 5	0.030	0.030	0.030
**Calculated analysis**			
AMEn (Kcal/kg)	2926	3010	3066
Crude protein	23.81	21.47	19.77
Calcium	0.934	0.740	0.642
Available Phosphorus	0.490	0.414	0.356
Sodium	0.177	0.177	0.178
Chloride	0.177	0.177	0.178
Potassium	0.860	0.748	0.667
DCAB (mEq/kg)	247	218	198
SID Lysine	1.298	1.164	1.068
SID Methionine	0.706	0.650	0.607
SID Methionine + Cyst(e)ine	0.983	0.907	0.850

Abbreviations: **DCAB**, Dietary cation-anion balance.; **SID**, Standardized ileal digestible.

1 Provided vitamins and minerals per kg diet: Vitamin A, 11000 IU; Vitamin D3, 4500 IU; Vitamin E, 60 IU; Vitamin K3, 3 mg; Vitamin B1, 3.5 mg; Vitamin B2; 8 mg; Vitamin B3; 19.5 mg; Vitamin B5; 60 mg; Vitamin B6, 4.55 mg; Vitamin B9; 2.2 mg; Vitamin B12; 0.022 mg; Biotin, 0.18 mg; Antioxidant, 20 mg; MnSO_4_, 120 mg; Zn (ZnSO_4_), 80 mg; Zn (ZnO), 40 mg; FeSO_4_, 30 mg; CuSO_4_, 16 mg; KIO_3_, 1.25 mg; Na₂SeO₃, 0.33 mg

2 Birds received the coccidiostat Salinomycin from day 1 to 28 and Maduracarb Rooyan™ from day 29 to 42 (Rooyandarou Co., Iran).

3 Phytase 10000 MicroTech VTR (VTR Biotech Co., China).

4 Zarin Binder (Vivan co., Iran).

5 The basal diets contained 0.03 % of sand, which was replaced by different types of protected butyrate (PB-A, PB-B, PB-C, or PB-D) to formulate the respective diets.

Birds were reared in 25 floor pens in an environmentally controlled poultry house. Each pen was provided with a 10-cm layer of paper bedding and equipped with one bell drinker and one tube feeder. Feed and drinking water were provided *ad libitum* throughout the experiment. During the first week, the lighting programme consisted of 23 h light and 1 h darkness daily, followed by 20 h light and 4 h darkness from d 8 to 42. Ambient temperature was maintained at 32°C during the first day and gradually reduced to 22°C by day 28. Relative humidity was maintained at 60-70% during the first 14 days and 45-50% thereafter until the end of the experiment. Based on these environmental conditions, the temperature-humidity index (THI) was calculated according to Dedousi et al. (2023) and is presented in Fig. 2. The poultry house was mechanically ventilated to maintain appropriate air quality and minimise heat accumulation. Birds were inspected at least twice daily, and mortality and general health status were recorded throughout the study. Birds were vaccinated according to the routine vaccination program used in the region. No therapeutic medications were administered during the experiment.

**Fig. 2:**
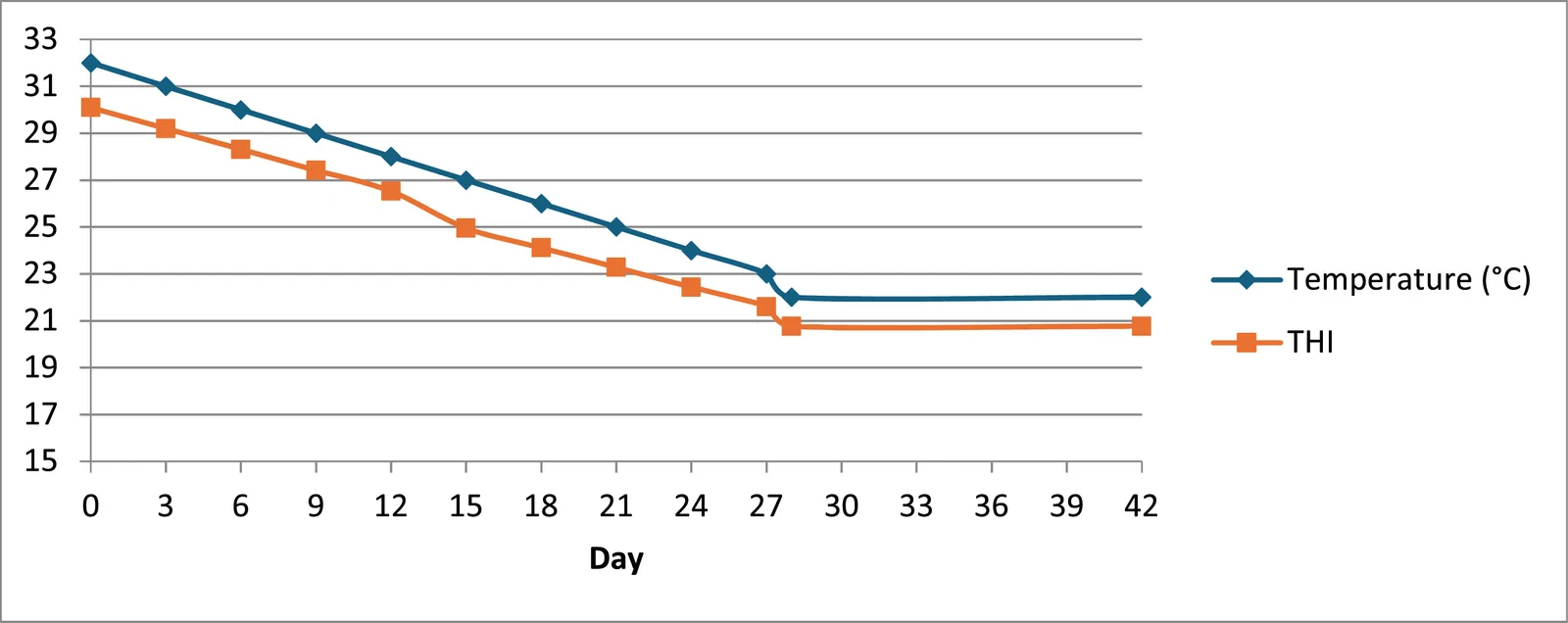
Temperature and temperature-humidity index (THI) throughout the 42-day experimental period.

### 2.3 Growth performance and carcass characteristics

On a pen basis, broiler body weight at the beginning and end of each phase, as well as feed consumption during each phase, were recorded throughout the study. Based on the recorded data, feed intake (FI), body weight gain (BWG), and feed conversion ratio (FCR) were determined. Overall performance was also evaluated across the entire experimental period.

At 42 days of age, two birds with body weights close to the mean body weight of their respective pen were selected from each replicate. Blood samples were collected via the jugular vein, after which the birds were euthanized and slaughtered. Following slaughter, the birds were defeathered, skinned, and eviscerated, and the skinless eviscerated carcass was weighed. Carcass yield was calculated relative to preslaughter live body weight. The weights of the breast, thigh, wings, back and neck, and feet were expressed as percentages of the skinless eviscerated carcass weight, whereas the weights of the proventriculus, gizzard, abdominal fat pad (AFP), heart, liver, spleen, bursa of Fabricius (BF), and small intestine were expressed as percentages of preslaughter live body weight. These birds (10 per treatment) were further used for intestinal morphology, serum biochemistry, and meat quality analyses.

### 2.4 Intestinal morphology

On day 42, intestinal samples (1 cm long) from mid-portions of the duodenum, jejunum, and ileum of two birds per replicate and preserved in 10% formalin for 24 h. Formalin-fixed tissue samples were then subjected to histological techniques, embedded in paraffin blocks, sectioned at 5 μm thickness, and stained with hematoxylin and eosin. The stained sections were microscopically observed using a light microscope (Olympus CX31, Olympus Corporation, Shinjuku, Tokyo, Japan). Ten well-oriented villi with intact lamina propria per bird were identified for quantification. Images were captured at 40 × magnification and analyzed using Image-Pro Plus software, version 4.5. Villus height (VH) was measured from the villus tip to the villus–crypt junction, crypt depth (CD) from the base of the crypt to the villus–crypt junction, and villus width (VW) at the midpoint of the villus. Villus surface area (VSA) was calculated using the equation: VSA = 2π × (VW/2) × VH (Al Sulaiman and Abudabos, 2026). Fig. 3 presents representative micrographs of the duodenal, jejunal, and ileal mucosa of broiler chickens at 42 days of age, obtained at 40 × magnification.

**Fig. 3:**
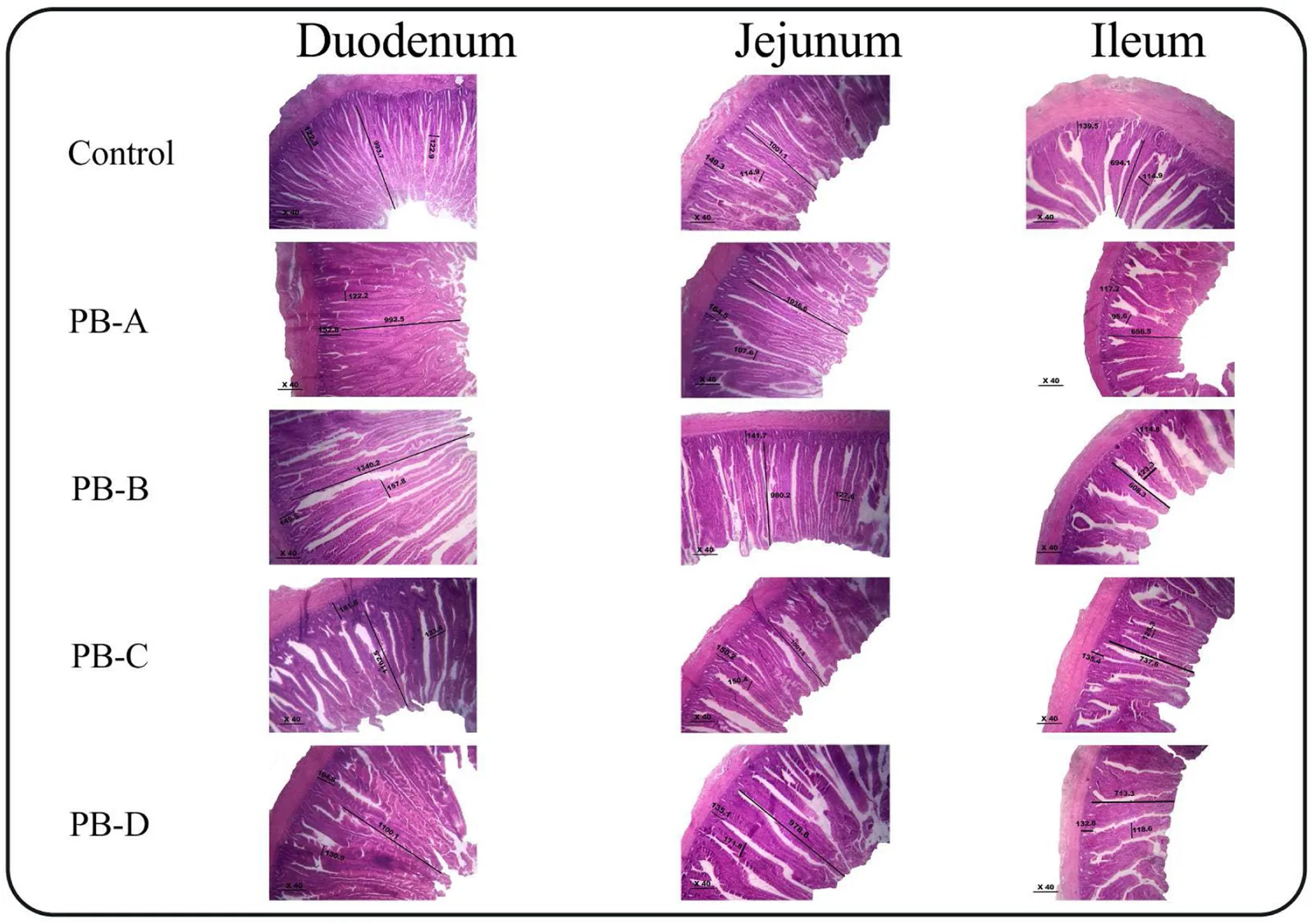
Histological features of the duodenal, jejunal, and ileal mucosa of 42-day-old broiler chickens based on hematoxylin and eosin staining. Images are presented at 40 × magnification. Abbreviations: **PB-A,** protected butyrate ButiPEARL®, Kemin; **PB-B,** butyrate protected with Eudragit® L100 copolymer; **PB-C,** butyrate protected with saturated fat; **PB-D,** butyrate protected with saturated fat and Tween 80.

### 2.5 Serum biochemistry

On day 42, the blood of two birds from each replicate pen (the same birds that were used for carcass analysis) before slaughter was sampled, and serum was prepared via centrifugation at 3200 × g for 15 min. The levels of cholesterol, total protein, albumin, ALT, AST, and ALP in the serum of chickens were measured using commercial test kits (Deltadarman Co., Tehran, Iran) on an autoanalyzer (Technicon RA-XT, Oakland, CA, USA). The level of MDA and the activity of SOD and CAT enzymes were determined using commercial test kits (Karmania Pars Gene, Kerman, Iran).

### 2.6 Meat quality

On day 42, the same birds used for carcass evaluation were also utilized for meat quality assessment. The left thigh muscle was collected for determination of meat quality traits. Meat quality parameters, including pH, water-holding capacity (WHC), meat cooking loss, and MDA concentration, were evaluated. About 1 g of raw thigh meat was homogenized and then, pH was measured with a digital pH meter (Shimaz Company, Tehran, Iran). WHC and cooking loss were determined according to (Ghiasvand et al., 2025). WHC was determined by placing a 1-g muscle sample on tissue paper in a centrifuge tube and centrifuging it at 1,500 × *g* for 4 min. The centrifuged sample was subsequently dried at 70°C for 24 hours. WHC was calculated as follows: WHC (%) = [(weight after centrifugation - weight after drying)/initial sample weight] × 100. Cooking loss was measured using 4-g thigh muscle samples. After recording their initial weights, the samples were placed in sealed bags and heated in a water bath at 70°C for 60 min and subsequently cooled under running water for 30 min. The cooked samples were then reweighed, and cooking loss was calculated as follows: cooking loss (%) = [(initial weight − cooked weight)/initial weight] × 100. MDA concentration was assessed using a modified thiobarbituric acid (TBA) method on meat samples stored at −20°C for 2 months (Ahammad and Kim, 2024; Sharifi et al., 2025).

### 2.7 Physical pellet quality (PPQ)

The experimental diets were prepared separately for each treatment. The ingredients were weighed and thoroughly mixed, and the respective PB product was incorporated into each treatment diet. The mash diets were processed in a double-barrel conditioner using short-term conditioning for 40 s at a steam pressure of 70 psi and a discharge temperature of 65°C. The conditioned diets were pelleted using a pellet mill equipped with dies of 2, 3, and 4 mm diameter for the starter, grower, and finisher diets, respectively. Following pelleting, the diets were cooled to approximately room temperature. Four independent batches of pelleted finisher feed were produced separately for each dietary treatment. One 500-g sample was collected from the middle portion of each batch, sieved through a 3.150-mm analytical sieve (Eckhardt DIN 4188, Haan, Germany), and stored in a labelled nylon bag until analysis. Thus, the independently produced feed batch was considered the experimental unit for pellet durability index (PDI) analysis, with four replicates per treatment. Briefly, clean pellet samples were subjected to a Pfost tumbling box (10 min at 50 rpm) according to the procedure reported by Mohammadi Ghasem Abadi et al. (2019). The PDI was calculated using the following formula: PDI (%) = (final intact pellet weight / initial pellet weight) × 100.

### 2.8 Statistical analysis

Statistical analyses were conducted in Minitab 18 (Minitab) using the general linear model (GLM) procedure appropriate for a completely randomized design. The model used was Yij = μ + Ti + eij, where Yij is the observed response, μ is the mean of treatment, Ti is the effect of dietary treatment, and eij is the error associated with each measurement. The normality of the data was checked using the Shapiro–Wilk test before doing any statistical analysis. All animal-related variables were analyzed using the pen as the experimental unit. For variables involving multiple observations within the same pen, the observations were averaged to obtain a single value before statistical analysis. For PDI, each independently prepared feed batch was considered the experimental unit, with four batches per dietary treatment. Treatment means were compared using Tukey’s multiple range test, with statistical significance set at *P* ≤ 0.05.

## 3 Results

### 3.1 Growth performance

Table 2 summarizes the growth performance responses of broilers to dietary treatments. During the starter phase, dietary treatment affected FI and BWG (*P* < 0.01). Compared with the control, PB-C reduced FI by 28.6% and BWG by 24.3%, whereas the other PB treatments did not differ significantly from the control. Starter FCR was also affected by dietary treatment (*P* < 0.05), with PB-B reducing FCR by 7.5% relative to the control. PB-A, PB-C, and PB-D had intermediate FCR values.

**Table 2:** Effect of dietary treatments on the growth performance of broilers during starter (d 1-14), grower (d 15-28), finisher (d 29-42) and overall (d 1-42) phases.

Item	Control	PB-A	PB-B	PB-C	PB-D	SEM	P-value
**FI (g/bird)**							
Starter	511 ^a^	506 ^a^	490 ^a^	365 ^b^	461 ^a^	16	0.0001
Grower	1450 ^a^	1392 ^ab^	1473 ^a^	1316 ^b^	1370 ^ab^	27	0.004
Finisher	2286	2271	2323	2257	2224	60	0.82
Overall	4247 ^ab^	4170 ^ab^	4286 ^a^	3938 ^b^	4056 ^ab^	82	0.04
**BWG (g/bird)**							
Starter	378 ^a^	389 ^a^	391 ^a^	286 ^b^	357 ^a^	14	0.0001
Grower	979 ^a^	964 ^a^	966 ^a^	863 ^b^	932 ^ab^	19	0.004
Finisher	1240	1207	1241	1243	1212	48	0.96
Overall	2597	2560	2598	2398	2502	64	0.18
**FCR**							
Starter	1.352 ^a^	1.302 ^ab^	1.251 ^b^	1.277 ^ab^	1.294 ^ab^	0.02	0.01
Grower	1.481	1.446	1.525	1.521	1.469	0.04	0.53
Finisher	1.844	1.883	1.878	1.827	1.834	0.04	0.79
Overall	1.635	1.628	1.650	1.649	1.621	0.03	0.92

^a-b^ Means within a row without common superscript are significantly different (P < 0.05).

Abbreviations: **FI**, feed intake; **BWG**, body weight gain; **FCR**, feed conversion ratio; **PB-A,** protected butyrate ButiPEARL®, Kemin; **PB-B,** butyrate protected with Eudragit® L100 copolymer; **PB-C,** butyrate protected with saturated fat; **PB-D,** butyrate protected with saturated fat and Tween 80.

During the grower phase, dietary treatment affected FI and BWG (P < 0.01). Compared with the control, PB-C reduced FI by 9.2% and BWG by 11.8%. Grower FCR was not affected by dietary treatment (*P* > 0.05). During the finisher period, no significant differences were noted for FI, BWG, and FCR due to dietary treatments (*P* > 0.05). Over the entire experimental period, FI was affected by treatment (*P* < 0.05), with PB-C showing an 8.1% lower value than PB-B. However, FCR and BWG were not affected by dietary treatments (*P* > 0.05).

#### 3.1.1 Carcass characteristics

Table 3 shows the impact of diets on carcass traits of broiler chickens. Dietary treatments did not significantly affect the relative weights of the carcass, breast, thigh, wings, back and neck, proventriculus, gizzard, heart, liver, spleen, bursa of Fabricius, small intestine, or feet (*P* > 0.05). Significant difference was only seen in case of AFP (*P* < 0.05). The birds that were offered PB-C diet had significantly more AFP than those offered PB-A and PB-B, while the control and PB-D treatments showed intermediate values.

**Table 3:** Effects of dietary treatments on carcass yield (% of preslaughter live body weight), carcass-part weights (% of skinless eviscerated carcass weight), and internal-organ weights (% of preslaughter live body weight) in broiler chickens.

Item	Control	PB-A	PB-B	PB-C	PB-D	SEM	P-value
Carcass	63.4	63.4	63.7	59.1	63.2	1.38	0.13
Breast	42.6	39.4	40.3	39.5	38.5	1.17	0.16
Thigh	28.8	31.2	30.4	31.0	30.1	0.88	0.36
Wings	8.0	8.1	8.5	8.1	8.3	0.19	0.52
Back and neck	20.3	21.2	20.7	21.1	23.0	0.84	0.25
Proventriculus	0.89	0.81	0.85	0.90	0.91	0.09	0.94
Gizzard	1.12	1.26	1.22	1.38	1.16	0.10	0.47
AFP	2.96 ^ab^	2.33 ^b^	2.45 ^b^	3.23 ^a^	2.76 ^ab^	0.21	0.04
Heart	0.93	0.90	1.07	1.02	0.96	0.08	0.68
Liver	3.9	4.0	4.1	4.3	4.1	0.29	0.86
Spleen	0.20	0.25	0.26	0.34	0.26	0.04	0.41
BF	0.08	0.14	0.16	0.20	0.12	0.03	0.22
Small intestine	8.8	9.0	8.8	9.1	8.7	0.56	0.98
Feet	5.8	6.1	6.0	6.3	6.0	0.19	0.40

^a-b^ Means within a row without common superscript are significantly different (P < 0.05).

Abbreviations: **AFP**, abdominal fat pad; **BF**, bursa of Fabricius; **PB-A,** protected butyrate ButiPEARL®, Kemin; **PB-B,** butyrate protected with Eudragit® L100; **PB-C,** butyrate protected with saturated fat; **PB-D,** butyrate protected with saturated fat and Tween 80.

### 3.2 Intestinal morphology

Table 4 summarizes the intestinal morphological responses to dietary treatments at 42 days of age. In the duodenum, significant differences were observed among treatments for VH, CD, VW, VH: CD, and VSA (*P* < 0.05). All PB-supplemented treatments showed increases in VH and VH:CD compared with the control group, with PB-B significantly increasing these measurements by 35.5% and 40.6%, respectively (*P* < 0.05). CD responded significantly to dietary treatments (*P* < 0.05), with birds fed PB-B showing a significantly lower value than those fed PB-D. VW was significantly higher in birds receiving PB-D versus the control group (P < 0.05), while other treatments showed intermediate values. VSA differed significantly among treatments, with PB-D producing the highest value and increasing VSA by 84.2% relative to the control (*P* < 0.01), while the other PB-supplemented groups had intermediate values. In contrast, no significant differences were detected among dietary treatments in jejunal or ileal morphology, including VH, CD, VW, VH:CD, and VSA (*P* > 0.05).

**Table 4:** Effect of dietary treatments on intestinal morphology on day 42.

Item	Control	PB-A	PB-B	PB-C	PB-D	SEM	P-value
**Duodenum**							
VH, μm	994 ^b^	1101 ^ab^	1347 ^a^	1164 ^ab^	1273 ^ab^	76.6	0.03
CD, μm	155 ^ab^	165 ^ab^	150 ^b^	162 ^ab^	188 ^a^	8.0	0.02
VW, μm	124 ^b^	131 ^ab^	159 ^ab^	134 ^ab^	181 ^a^	13.8	0.04
VH:CD	6.4 ^b^	6.7 ^ab^	9.0 ^a^	7.1 ^ab^	6.8 ^ab^	0.59	0.03
VSA, μm^2^	390821 ^b^	452728 ^ab^	689693 ^ab^	493005 ^ab^	720087 ^a^	71167	0.01
**Jejunum**							
VH, μm	1002	1037	981	1002	979	74.2	0.98
CD, μm	150	164	142	150	172	13.6	0.54
VW, μm	116	108	128	151	136	15.6	0.35
VH:CD	6.8	7.0	6.9	6.7	5.7	0.86	0.81
VSA, μm^2^	364392	351558	400207	477180	437793	63447	0.61
**Ileum**							
VH, μm	695	657	609	738	714	51	0.44
CD, μm	140	118	115	136	133	7.5	0.10
VW, μm	116	96	124	126	119	8.8	0.18
VH:CD	5.0	5.5	5.3	5.4	5.3	0.31	0.82
VSA, μm^2^	253928	201135	241447	291317	272121	29873	0.30

^a-b^ Means within a row without common superscript are significantly different (P < 0.05).

Abbreviations: **VH**, villus height; **VW**, villus width; **CD**, crypt depth; **VH:CD**, villus height to crypt depth ratio**; VSA**, villus surface area; **PB-A,** protected butyrate ButiPEARL®, Kemin; **PB-B,** butyrate protected with Eudragit® L100 copolymer; **PB-C,** butyrate protected with saturated fat; **PB-D,** butyrate protected with saturated fat and Tween 80.

### 3.3 Serum biochemistry

Results of the impacts of diet-based treatments on biochemical profile of serum in birds at day 42 are depicted in Table 5. All values of cholesterol, total protein, albumin, ALT, AST, ALP, MDA, and SOD activity remained comparable across the dietary groups (*P* > 0.05). However, catalase activity showed a trend toward being lower in PB-D treated birds compared to control group (*P* = 0.08).

**Table 5:** Effect of dietary treatments on serum biochemistry on day 42.

Item	Control	PB-A	PB-B	PB-C	PB-D	SEM	P-value
Cholesterol, mg/dL	148	154	158	148	163	14	0.93
Protein, g/dL	3.0	3.0	3.5	3.6	3.8	0.30	0.29
Albumin, g/dL	1.1	1.1	1.3	1.4	1.5	0.12	0.12
ALT, u/L	16.5	15.5	21.3	19.6	17.8	3.7	0.81
AST, u/L	339	365	302	354	413	48	0.59
ALP, u/L	3689	4779	4199	3661	4112	669	0.76
MDA, nmol/mL	7.2	12.5	8.7	12.0	8.0	2.74	0.67
SOD, U/mL	8.9	10.8	10.3	8.1	9.5	2.0	0.90
Catalase, U/mL	405	348	326	316	272	30.8	0.08

Means within a row without superscript letters are not significantly different (P > 0.05).

Abbreviations: **ALT**, alanine aminotransferase; **AST**, aspartate aminotransferase; **ALP**, alkaline phosphatase; **MDA**, malondialdehyde; **SOD**, superoxide dismutase; **PB-A,** protected butyrate ButiPEARL®, Kemin; **PB-B,** butyrate protected with Eudragit® L100 copolymer; **PB-C,** butyrate protected with saturated fat; **PB-D,** butyrate protected with saturated fat and Tween 80.

### 3.4 Meat quality

Table 6 summarizes the meat quality responses to the dietary treatments at 42 days of age. Both pH and MDA content remained unaffected by the dietary treatments (*P* > 0.05). However, WHC was significantly higher in birds fed PB-A, PB-B, and PB-D than in those receiving PB-C, representing increases of 2.3%, 2.3%, and 2.5%, respectively (*P* < 0.01), and was numerically greater than in the control group. Meat cooking loss was significantly reduced by 12.6% and 9.5% in birds fed PB-B and PB-D, respectively, compared with the control (*P* < 0.01).

**Table 6:** Effect of dietary treatments on meat quality on day 42.

Item	Control	PB-A	PB-B	PB-C	PB-D	SEM	P-value
pH	5.7	5.6	5.6	5.8	5.7	0.10	0.88
WHC, %	64.7 ^ab^	65.5 ^a^	65.5 ^a^	64.0 ^b^	65.6 ^a^	0.31	0.007
Cooking loss, %	42.2 ^a^	41.9 ^ab^	36.9 ^c^	39.5 ^abc^	38.2 ^bc^	0.87	0.001
MDA, nmol/g	1.20	1.22	1.15	1.25	1.20	0.03	0.38

^a-c^ Means within a row without common superscript are significantly different (P < 0.05).

Abbreviations: **WHC**, water-holding capacity; **MDA**, malondialdehyde; **PB-A,** protected butyrate ButiPEARL®, Kemin; **PB-B,** butyrate protected with Eudragit® L100 copolymer; **PB-C,** butyrate protected with saturated fat; **PB-D,** butyrate protected with saturated fat and Tween 80.

### 3.5 Physical pellet quality (PPQ)

Fig. 4 illustrates the effects of dietary treatments on the PDI of finisher feed. Dietary treatments significantly affected the PDI of the feed (*P* < 0.001). The treatment PB-A had the highest PDI at 76.9%, followed by PB-C and PB-D, which were also relatively high at 76.6% and 75.8%, respectively. The results indicate that the PDI values for PB-A, PB-C, and PB-D treatments were significantly higher than those for the control (71.4%) and PB-B (67.0%) (*P* < 0.05). The PB-B treatment produced the lowest PDI and was significantly lower than all other treatments. No significant difference was observed between PB-A, PB-C, and PB-D.

**Fig. 4:**
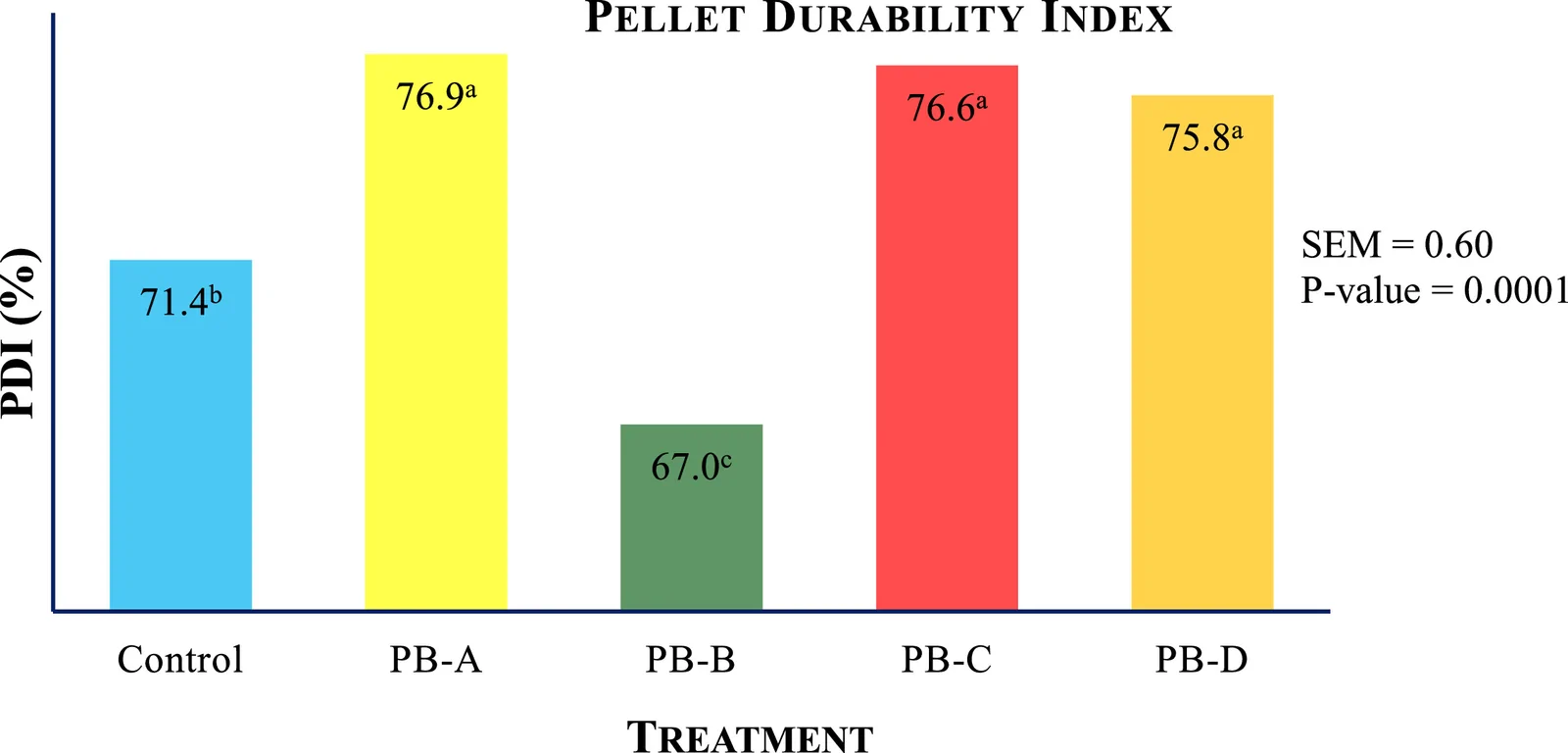
Effect of dietary treatments on finisher feed pellet durability index. ^a-c^ Means without common superscript are significantly different (P < 0.05). Abbreviations: **PB-A,** protected butyrate ButiPEARL®, Kemin; **PB-B,** butyrate protected with Eudragit® L100 copolymer; **PB-C,** butyrate protected with saturated fat; **PB-D,** butyrate protected with saturated fat and Tween 80.

## 4 Discussion

The present study examined the responses of broiler chickens fed PBM-containing diets to different PB formulations, with PBM representing a relatively variable and less digestible alternative protein source. Overall, the responses varied according to the PB coating system, with the most consistent effects observed in early growth performance, intestinal morphology, meat quality, and PPQ rather than overall growth performance.

The improvement in starter-phase FCR observed in PB-supplemented birds is consistent with previous studies reporting beneficial effects of protected or coated butyrate during early growth and intestinal development in broiler chickens (Ghasemi et al., 2025; Kaczmarek et al., 2016; Pires et al., 2023). Specifically, Kaczmarek et al. (2016) found that broiler chicken dietary supplementation with 0.3 and 0.4 g/kg of PB (ButiPEARL®, Kemin) resulted in better FCR than the control treatment during the first two weeks. Protected butyric acid provides energy to intestinal epithelial cells and may enhance mucosal integrity, suppress pathogenic bacteria, and improve nutrient absorption, ultimately contributing to better FCR in challenged birds (Khan et al., 2022; Mátis et al., 2022; Sadurní et al., 2022). The superior response of PB-B may be attributed to its pH-sensitive Eudragit® L100 coating, which enables targeted release in the small intestine, thereby increasing butyrate availability at the site of maximal absorption and epithelial activity (Jain et al., 2020; Sester et al., 2020). The observed improvement in early FCR indicates that PB-B enhanced feed efficiency during a physiologically sensitive stage when the gastrointestinal tract is still developing.

Interestingly, birds receiving PB-C exhibited lower FI throughout the experiment, which may have contributed directly to the reduction in BWG. The saturated fat matrix used in this treatment contained a high proportion of palmitic acid and may have altered both the additive release pattern and sensory characteristics. Although the exact mechanism remains unclear, the reduction in FI may have resulted from differences in palatability or butyrate release from the saturated-fat matrix. Because feed palatability and gastrointestinal release kinetics were not measured, these potential mechanisms cannot be distinguished. These findings emphasize that the efficacy of protected butyrate depends not only on the amount supplied but also on the characteristics of the coating system and its release behavior within the gastrointestinal tract (Gavali et al., 2026; Moquet et al., 2015; Moquet et al., 2016). Across published studies, the effects of protected butyrate (PB) on growth performance throughout the entire production cycle have been inconsistent. Several studies have reported beneficial effects primarily during the early stages of growth (Lan et al., 2020; Pires et al., 2023). In contrast, Wu et al. (2018) observed no significant differences in growth performance throughout the 42-day feeding period, suggesting that the growth-promoting effects of butyric acid may decline as birds age, in line with our findings. Additionally, Ghasemi et al. (2025) demonstrated that protected butyric acid markedly reduced the adverse impacts of coccidiosis, such that performance in broiler chickens were restored to levels similar to those of the unchallenged negative control. However, because no disease challenge was applied in the present study, these findings cannot be directly compared with our results.

Most carcass characteristics were not significantly affected by PB supplementation, in agreement with earlier reports indicating that protected butyrate generally have limited effects on carcass yield parameters (Ghasemi et al., 2025; Pires et al., 2023). Regarding AFP, the present results are inconsistent with those of Sadurní et al. (2022), who found that supplementing broiler diets with sodium butyrate protected by medium-chain fatty acid salts did not alter AFP. A clear response to PB supplementation was observed in duodenal morphology, particularly in PB-B treatment, which improved VH and VH:CD compared with the control group. All PB-supplemented treatments numerically increased VSA compared with the control group. These changes are indicative of enhanced intestinal health, improved absorptive surface area, and reduced epithelial turnover (Amiri et al., 2019). Butyrate has been shown to regulate gene expression related to tight junction proteins and mucosal integrity, thereby reinforcing gut barrier function and facilitating more efficient nutrient uptake (Elnesr et al., 2020; Ghasemi et al., 2025). The improvement in villus architecture observed in the present study is consistent with reports showing that PB increases VH and improves gut morphology in broilers under both normal and challenged conditions (Kaczmarek et al., 2016; Mátis et al., 2022). The absence of significant effects in jejunum and ileum suggests that the release site of PB formulations was primarily in the proximal intestine, or that the duodenum is more responsive to butyrate supplementation under diets containing alternative protein sources such as PBM. This regional specificity has been previously reported for coated butyrate preparations (Panda et al., 2009).

Animal physiological and metabolic status can be reflected by serum biochemical indices, which are strongly influenced by environmental and nutritional factors. Total protein and albumin are key indicators of protein metabolism and provide insight into the balance between protein synthesis and degradation in broiler chickens (Hou et al., 2024). Likewise, AST, ALT, and ALP are important biochemical markers commonly used to assess liver function, hepatocellular integrity, and metabolic status in poultry. Alterations in the serum activities of these enzymes may reflect changes in hepatocellular integrity and liver metabolism (Alimohammadi et al., 2024; Selim et al., 2021). Oxidative status is an important physiological marker, as excessive generation of reactive oxygen species (ROS) can exceed endogenous antioxidant capacity and induce oxidative stress. Antioxidant enzymes such SOD and CAT, along with lipid peroxidation markers such as MDA, are commonly used to assess the balance between oxidant production and antioxidant defense in broilers (Gu et al., 2025).

Overall, the serum biochemical profile observed in our study is in line with previous study (Gu et al., 2025), showing that dietary butyrate supplementation often exerts limited effects on serum protein fractions and hepatic enzyme activities in broilers, although responses may vary depending on the chemical form, coating technology, and release characteristics of the additives. In contrast to the present findings, Gu et al. (2025) reported increased SOD and CAT activities accompanied by reduced MDA levels in broilers fed coated sodium butyrate, indicating an enhanced antioxidant status. In the current study, a numerical but non-significant reduction in catalase activity was observed in birds fed PB-D. Given the central role of catalase in hydrogen peroxide detoxification, this tendency may suggest a slight reduction in enzymatic antioxidant capacity; however, due to the lack of statistical significance, this response should be interpreted with caution.

The higher WHC observed in the PB-A, PB-B, and PB-D groups relative to PB-C, together with the reduced cooking loss in birds fed PB-B and PB-D compared with the control, was generally consistent with the findings of Lan et al. (2020). These researchers found that the inclusion of broiler chicken diets with 300–1200 ppm of the sodium butyrate improved meat quality, as indicated by numerically lower cooking loss and significantly reduced drip loss. These results are also in line with Zhang et al. (2011), who found that dietary sodium butyrate supplementation significantly decreased 24-h drip loss in corticosterone-challenged broilers. In the current experiment, a numerical reduction in muscle MDA content was observed in the PB-B group compared with the other treatments. This trend is in agreement with the findings of Lan et al. (2020) and Zhang et al. (2011), who reported that sodium butyrate supplementation reduced lipid peroxidation in breast muscle.

Pelleting is the most widely used thermal processing technique in poultry feed manufacturing (Abdollahi et al., 2013). It improves feed handling characteristics, enhances production efficiency, and reduces feed wastage (You et al., 2025). However, achieving optimal pellet quality is often challenging due to the influence of multiple factors, including diet formulation, processing conditions, and other variables (You et al., 2025). A novel and practically important finding of this study is the significant effect coating materials of PB on PDI. All Lipid-based coatings of PB-A, PB-C, and PB-D improved pellet quality. Lipid-based coatings, particularly saturated fats e.g. palmitic acid, which had similar melting points (< 65°C) near to conventional temperature of pelleting process, may enhance pellet integrity by improving lubrication during pelleting and reducing fines formation (Mohammadi Ghasem Abadi et al., 2019; Zimonja et al., 2007). However, because changes in the coating materials during pelleting were not directly evaluated, this mechanism remains speculative.

The findings should be interpreted considering several limitations. Only one inclusion level of each PB product was evaluated, and gastrointestinal release kinetics and butyrate concentrations were not measured. Moreover, PPQ was assessed only in the finisher diets under a single set of pelleting conditions. Further studies evaluating dose-dependent responses and the release behavior of different coatings are therefore warranted.

## 5 Conclusion

Protected butyrate produced coating-dependent responses in broilers fed PBM-containing diets. PB-B improved starter FCR, duodenal morphology, and meat cooking loss, whereas lipid-coated forms enhanced pellet durability. However, PB-C reduced early growth performance. These findings are limited to a single PB inclusion level, and the intestinal release kinetics of the products were not directly evaluated.

## Declaration of generative AI use

ChatGPT (GPT-5; OpenAI) was used to assist in generating design elements for the graphical abstract. The final image was reviewed and edited by the authors, who take full responsibility for its content.

### Animal subject

This study was conducted in accordance with the following guidelines for animal welfare and/or reporting: the Research Animal Ethics Committee of the Faculty of Animal Science, Gorgan University of Agricultural Sciences and Natural Resources, Iran. This study was approved by the 20241506.

## CRediT authorship contribution statement

**Seyed Ghasem Shirangi:** Writing – review & editing, Project administration, Methodology, Investigation, Formal analysis. **Mahmoud Shams Shragh:** Writing – review & editing, Supervision, Methodology, Investigation, Conceptualization. **Behrouz Dastar:** Writing – review & editing, Methodology, Investigation, Conceptualization. **Mohammad Hossein Mohammadi:** Writing – review & editing, Methodology, Investigation, Formal analysis. **Enayat Rahmatnejad:** Writing – review & editing, Writing – original draft, Methodology, Investigation, Conceptualization.

## Declaration of competing interest

There are no potential conflict of interest regarding this manuscript.
